# Analysis of clinical application effects of Esketamine combining Sufentanil in labor analgesia and their impacts on postpartum depression

**DOI:** 10.12669/pjms.40.9.8689

**Published:** 2024-10

**Authors:** Haoyu Jiang, Shoubo Quan, Yahai Su, Jingyi Li, Xuefeng Zhang

**Affiliations:** 1 Haoyu Jiang, Department of Anesthesiology, The Affiliated Dongguan Songshan Lake Central Hospital, Guangdong Medical University, Dongguan 441000, Guangdong, China; 2 Shoubo Quan, Department of Anesthesiology, SSL Central Hospital of Dongguan City, Affiliated Shilong People’s Hospital of Southern Medical University, Dongguan 523326, Guangdong, China; 3 Yahai Su, Department of Anesthesiology, SSL Central Hospital of Dongguan City, Affiliated Shilong People’s Hospital of Southern Medical University, Dongguan 523326, Guangdong, China; 4 Jingyi Li, Department of Pain, SSL Central Hospital of Dongguan City, Affiliated Shilong People’s Hospital of Southern Medical University, Dongguan 523326, Guangdong, China; 5 Xuefeng Zhang, Department of Pain, SSL Central Hospital of Dongguan City, Affiliated Shilong People’s Hospital of Southern Medical University, Dongguan 523326, Guangdong, China

**Keywords:** Esketamine, Sufentanil, Labor analgesia, Clinical effect, Postpartum depression

## Abstract

**Objective:**

To evaluate the clinical effects of Esketamine combining Sufentanil in labor analgesia and their impacts on postpartum depression.

**Methods:**

This was a retrospective study. One hundred and fifty primiparae with spontaneous labor were selected at SSL Central Hospital of Dongguan City from July 10, 2021 to May 10, 2022 as the research objects and randomly divided into two groups (each n=75). While the control group underwent epidural infusion of Sufentanil for analgesia, the study group was administered Esketamine combining Sufentanil. Compared analgesic effects, time of birth course, adverse reactions, pain intensity, scores assigned to depression and anxiety before analgesia and after delivery were made for pregnant women in both groups.

**Results:**

The response rates of the study and control groups reached 100% and 93%, respectively, with statistically significant differences (p=0.02). The time of the first, second, and third stages of labor in the study group were all significantly lower than that of the control group; showing statistical significance (p=0.00). Regarding adverse reactions showed no statistically significant differences (p=0.44). Moreover, the study group showed noticeably lower pain intensity than those of the control group during delivery, 20 minutes and one hour after delivery; and their differences were statistically significant (p=0.00). After delivery, both the SAS and SDS of the study group were respectively lower than those of the control group, with statistically significant differences as well (p=0.00).

**Conclusions:**

Applying Esketamine combining Sufentanil in epidural painless delivery produces rather favorable analgesic effects, shortens the time of the birth process, and improves postpartum anxiety and depression without leading to more adverse reactions.

## INTRODUCTION

Labor and delivery are a process where the fetus separates itself from the mother and becomes an independent individual. During delivery, especially spontaneous labor, pregnant women experience intense pain which leads to various endocrine responses and eventually causes vasoconstriction and labor extension.^1^ Postpartum depression is a depressive state developed after delivery. Its main onset period is within six weeks after delivery.^2^ Once a pregnant woman is attacked by postpartum depression, she suffers dysphoria, anxiety, irritability, and depression, and also can be highly strung.

The existing literature^3^ find that more intense pain experienced by a pregnant woman corresponds to a higher occurrence rate of postpartum depression. Painless labor, as an advanced medical technique easy to implement and safe and mature, has been widely applied in childbirth for the past few years.^4^,^5^ Through various measures, this technology can ease the pain for pregnant women during delivery. On the premise of ensuring the safety of the mother and the fetus, effective drugs are administered without influencing uterine contraction so as to hinder the transmission of pain nerves and achieve an effect of removing the labor pain.^6^ To alleviate the pain and emotional issues in the course of painless labor, and guarantee maternal and fetal safety, effective anaesthetic methods and drugs should be adopted clinically. In this aspect, epidural anesthesia is a commonly used anaesthetic method in clinics. It not only shows significant analgesic effects by blocking spine nerve roots and benumbing its region od dominance, but also has no major influences on infant and mother.^7^

As for Sufentanil, it is an opioid agent with the strongest analgesic effect compared with other analgesics. It is fast-acting and long-lasting.^8^ Esketamine is an isomer of ketamine, producing rather good anesthetic and analgesic effects^9^ and showing more binding affinity with N-methyl-D-aspartate (NMDA) receptors. Therefore, Esketamine is capable of promoting the release of brain-derived neurotrophic factors so as to improve postpartum depression and lower the suicide rate of women after delivery. This study intended to explore clinical effects of Esketamine combining Sufentanil in labor analgesia and their therapeutic effect of postpartum depression.

## METHODS

This was a retrospective study. One hundred and fifty primiparae received in SSL Central Hospital of Dongguan City were selected from July 10, 2021 to May 10, 2022 as the research object, and divided into two groups randomly, with each group consisting of 75 cases. Patient data were retrieved from the hospital information and electronic medical record systems, collected their various information of all patients. In the study group, participants were 22-33 years old (mean age: 26.45±4.81); and those of the control group were 23-33 as well, with the mean age of 27.03±4.41. Baseline data of research objects are balanced between both groups, indicating comparability of these data (p>0.05), as shown in Table-I below.

**Table-I:** Comparisons of general data between study and control groups (*χ̅*±*S*) n=75.

Targets	Study group	Control group	t/χ^2^	P
Age	26.45±4.81	27.03±4.40	0.77	0.43
Gestational weeks	39.36±0.53	39.25±0.69	1.09	0.28
Weight (kg)	72.83±5.44	72.32±5.26	0.58	0.56
Height (cm)	163.30±16.57	162.84±17.05	0.17	0.86

P>0.05.

### Ethical Approval:

The study was approved by the Institutional Ethics Committee of SSL Central Hospital of Dongguan City (No.: KY2023171; date: August 1, 2023), and written informed consent was obtained from all participants.

### Inclusion criteria:

- Lying-in women in singleton pregnancy in full term;
- Those with normal cephalopelvic indexes;
- Those with no other obstetric pathological factors;
- Those with no contraindications to anesthesia;
- Those showing no fetal distress in the uterus;
- Those who have been informed and signed the letter of consent;
- Those with complete clinical data;
- Those with no mental/cognitive abnormalities, able to cooperate to complete this study, and showing preferable treatment compliance;
- Those with no contraindications to drugs used in this study;
- Those conforming to principles of vaginal trial birth^10^

### Exclusion criteria:

- Lying-in women with severe endocrine diseases;
- Those with severe respiratory and circulation system diseases;
- Those with dysfunction of major organs such as liver and kidney;
- Those with mental or nerve system diseases, and unable to cooperate with researchers satisfactorily;
- Those taking drugs such as hormones and immunosuppressor recently that may affect this study.

After participants of both groups entered the labor room, their physical signs were strictly monitored, including blood pressure, blood oxygen, and fetal heart rate. A venous access was constructed, with patients lying down in the correct position. In an interval of L2 and L3, epidural puncturing was implemented, and a 3-4 cm long segment of the catheter indwelled. If no blood or cerebrospinal fluid was observed during withdrawal, analgesics should be administered accordingly. In the control group, an epidural pumped into Sufentanil was performed to ease their pain. To be concrete, Sufentanil in a dosage of 3μg/kg was mixed with 100ml of normal saline (NS), and their mixture was epidurally infused dropwise. In this scenario, the maintenance dose of continuous infusion was set at 6mL/h, the self-control dosage at 7ml per time, and the corresponding locking time at 30 minutes. In the study group, Esketamine combining Sufentanil was administered. That is, Esketamine in a dosage of 1mg/kg was combined with Sufentanil in a dosage of 2μg/kg and 100 ml of NS. As for infusion velocity and durations, they are the same as those established for the control group.

### Observation targets:

First, analgesic effects were compared between the groups before analgesic administration. The corresponding analgesic effect assessment standards are given below:^11^

- Zero indicates no pain during delivery;
- One, slight pain during delivery;
- Two, moderate pain during delivery and tolerable
- Three, intense pain during delivery and intolerable; and
- Four, extreme pain during delivery.

Moreover, scores of 0 and one signify that the drugs produce certain effect; two means that the drugs are effective; and three and four indicate inefficacy. Here, the Score of the Total Effective Rate= (Scores Assigned to Producing Effect and Being Effective)/The Total Number of Cases×100%. Second, the stages of labor and the time of each stage were compared. Third, adverse reactions occurring between the groups were compared, including skin pruritus, nausea, vomiting, and uroschesis. Fourth, pain intensity during delivery was compared. Here, their pain intensity was evaluated by the Visual Analog Scale(VAS) during analgesia, at 20 min after delivery, and at one hour after delivery. Through a comparative analysis of differences in their pain intensity, scores in a range of 0-10 were assigned. The higher the score is, the higher the pain degree will be.^12^ At last, anxiety scores before analgesia and after delivery were compared. Respectively, a self-rating anxiety scale (SAS) and a self-rating depression scale (SDS)^13^ were adopted to evaluate emotional variations before and after relevant interferences in both groups. The lower the score is, the better their mood will be.

### Statistical analyses:

SPSS 20.0 was selected for all data statistics, and relevant measurement data was denoted as(*χ̅*±*S*). For inter-group, intra-group, and pairwise comparisons, independent-samples T-test, variance analysis of repeatedly measured data, and T-test are respectively utilized. To compare various rates, χ^2^ test is selected. If P<0.05, it is deemed statistically significant.

## RESULTS

Results of comparative analyses on analgesic effects between the study and control groups are shown in Table-II. It is indicated that the effective rates of the study and control groups are 100.00% and 93%, respectively. As can be seen, the effective rate of the study group is significantly higher than that of the control group, and their differences are of statistical significance, p=0.02.

**Table-II:** Comparisons of analgesic effects between study and control groups (*χ̅*±*S*) n=75.

Groups	Producing certain effect	Being effective	Ineffective	Total effective rate
Study	68	7	0	75 (100.00%)
Control	61	9	5	70 (93%)
*χ* ^2^				5.17
*P*				0.02

*P<0.05.

As implied by comparative analyses on stages of labor between the study and control groups, the time taken by the first, second and third stages of labor of the former is prominently lower than that of the latter. Their differences show statistical significance, that is p=0.00 (Table-III).

**Table-III:** Comparisons of stages of labor between study and control groups (*χ̅*±*S*) n=75.

Groups	First stage of labor (min)*	Second stage of labor (min)*	Third stage of labor (min)*
Study	657.54±12.03	73.18±9.43	16.57±2.84
Control	712.57±20.75	96.70±11.26	24.66±3.47
t-value	18.26	12.54	13.97
P-value	0.00	0.00	0.00

* P<0.05.

Occurrence of adverse drug reactions are compared between the groups. According to corresponding results, ADR occurrence in the study and control groups were 13% and 9%, respectively. Clearly, the adverse drug reaction occurrence rate of the former is higher than that of the latter. However, their differences show no statistical significance (p=0.44), as listed in Table-IV.

**Table-IV:** Comparisons of adverse drug reaction occurrence rates between the study and control groups (*χ̅*±*S*) n= 75.

Groups	Respiratory depression	Skin pruritus	Gastrointestinal reactions	Uroschesis	Occurrence rate (%)
Study	0	3	5	2	10 (13%)
Control	0	2	4	1	7 (9%)
*χ* ^2^					0.60
P-value					0.44

p>0.05.

As the time after delivery is extended, VAS based scores are all apparently reduced in both groups (P=0.00). More particularly, that of the study group is significantly lowered compared with that of the control group during delivery, and at 20 min and one hour after delivery. In addition, their differences are statistically significant (P=0.00). Table-V.

**Table-V:** Comparisons of analgesic effects between the study and control groups (*χ̅*±*S*) n=75.

Groups	During delivery*	20 min after delivery*	1 hour after delivery*	F	P-value
Study*	4.27±0.22	3.36±0.17	1.57±0.10	12.48	0.02
Control*	4.69±0.35	3.55±0.24	1.74±0.21	13.63	0.00
t-value	7.87	5.03	5.67		
p-value	0.00	0.00	0.00		

* p<0.05.

SAS and SDS levels before delivery have no prominent differences between the study and control groups, that is P>0.05. However, such levels in the study group are more significantly reduced one week after delivery compared with those in the control group. Likewise, their differences are of statistical significance (p=0.00), as shown in Table-VI.

**Table-VI:** Comparisons of emotional status in the study and control groups after interferences (*χ̅*±*S*) n=75.

Targets		Study *	Control	t-value	p-value
SAS	Before analgesia	60.21±8.53	60.58±8.46	0.24	0.81
	After delivery*	51.45±7.62	55.49±6.85	3.05	0.00
SDS	Before analgesia	63.68±7.49	64.17±7.57	0.36	0.72
	After delivery*	51.03±6.85	56.21±6.44	4.27	0.00

* p<0.05.

## DISCUSSIONS

In our study, it is demonstrated that a combination medication of Esketamine and Sufentanil is superior to a single dosage of Sufentanil in their effective rates (i.e., 100% vs. 93%; p=0.02). The time consumed by the first, second, and third stages of labor in the study group is significantly shorter than that in the control group (p=0.00). As for adverse reactions, no significant ncreases are caused (p=0.44). Scores assigned to pain intensity drop to a greater degree in the study group compared with the control group (P=0.00). This implies that Esketamine combining Sufentanil can effectively ease uterine contraction inducing pain. By suppressing glutamate release in the presynaptic membrane, it enables the concentration of magnesium ions in the postsynaptic membrane to rise, so that NMDA receptor channels can be closed. In this way, it can fight against hyperpathia induced by opioids^14^ Therefore, combined medication of Esketamine and Sufentanil has a more significant suppressive action on contraction pain than the single dosage of Sufentanil. In this study, both SAS and SDS scores assigned to the study group are more obviously lowered compared with those in the control group (p=0.00). Considering onset of depression forms a correlation to target protein signal suppression of mammalian rapamycin, ketamine has the potential to indirectly promote brain-derived neurotrophic factor synthesis and release and rapidly activate target protein signaling of mammalian rapamycin by blocking NMDA receptors. In this way, the depressive state can be significantly improved in hours.^15^ In a word, combining Esketamine with Sufentanil produces a good therapeutic effect on postpartum depression. Tu et al.^16^ believed that Esketamine can block NMDA receptors and boost expression levels of brain-derived neurotrophic factors, thus relieving the depressive symptoms of lying-in women.

Effective anesthesia is generally adopted clinically to ease the pain during delivery and minimize negative impacts of labor pain on the mother and the infant without extending the stage of labor, thus assisting lying-in women in smooth delivery. In addition, favorable labor analgesia must have no prominent suppressions on uterine contraction on one hand, and does not cause arrested/prolonged labor or postpartum hemorrhage. This can ensure that lying-in women are sober and their pain can be removed, and fetal respiration and circulations will not be inhibited as well. In this aspect, epidural block-based anesthesia is a proved method.^17^

Sufentanil, as an opioid agent with analgesic effects, is derived from Fentanyl.^18^ It can easily penetrate through the blood brain barrier and produce significant analgesic effects. Moreover, the duration of its analgesic effect is longer than that of Fentanyl.^19^ Thanks to its low toxicity and minor respiratory inhibition actions, Sufentanil is commonly used for patient-controlled analgesia in clinics.^20^ Vora et al.^21^ compared clinical efficacy produced by Sufentanil and morphine used for analgesia after cesarean section, and found that VAS based scores assigned to lying-in women in the Sufentanil group are significantly lower than those in the morphine group. This reflects that Sufentanil alone can outperform morphine in its analgesic effects after cesarean section. In spite of being fast-acting once injected, its effective time is rather short. In most cases, it is less likely to achieve satisfactory treatment results, therefore, it is necessary to combine Sufentanil with other drugs to obtain better analgesic effects.

Esketamine is an isomer of ketamine, playing an effective role in sedation and analgesia. According to relevant research,^22^ Esketamine of a subanesthetic dose can exert analgesic effects, and has a dextral structure stronger with higher titer among ketamine. Featured with a short elimination half-life, rapid revival, slight respiratory suppression, and minimal adverse reactions in the mental system, most of Esketamine can be completely metabolized. Thus, it is applicable for anesthesia in minor surgery.

### Limitations:

It includes a small sample size, and a failure in incorporating other common narcotic drugs for comparative analyses. In future, we will increase the sample size, enrich details about follow-up visits, and include other narcotic drugs commonly used in clinics to more elaborately describe the advantages and disadvantages of this therapeutic regimen.

## CONCLUSIONS

Esketamine combining Sufentanil produces significant effects of epidural analgesia, shortens the labor process, relieves the pain during labor, improves anxiety and depression status, and reduces relevant adverse reactions, demonstrating significant analgesic effects.

### Authors’ Contributions:

**HJ** and **XZ**: Carried out the studies, data collection, drafted the manuscript, are responsible and accountable for the accuracy or integrity of the work.

**SQ YS JL:** Performed the statistical analysis and participated in its design and review.

Performed the statistical analysis and participated in its design. All authors read and approved the final manuscript.
